# A bacterial hemerythrin-like protein MsmHr inhibits the SigF-dependent hydrogen peroxide response in mycobacteria

**DOI:** 10.3389/fmicb.2014.00800

**Published:** 2015-01-15

**Authors:** Xiaojing Li, Jun Tao, Xinling Hu, John Chan, Jing Xiao, Kaixia Mi

**Affiliations:** ^1^CAS Key Laboratory of Pathogenic Microbiology and Immunology, Institute of Microbiology, Chinese Academy of SciencesBeijing, China; ^2^Departments of Medicine and Microbiology and Immunology, Albert Einstein College of MedicineBronx, NY, USA; ^3^Beijing Key Laboratory of Microbial Drug Resistance and ResistomeBeijing, China

**Keywords:** mycobacteria, hemerythrin-like protein, MsmHr, SigF, hydrogen peroxide

## Abstract

Hydrogen peroxide (H_2_O_2_) is one of a variety of reactive oxygen species (ROS) produced by aerobic organisms. Host production of toxic H_2_O_2_ in response to pathogen infection is an important classical innate defense mechanism against invading microbes. Understanding the mechanisms by which pathogens, in response to oxidative stress, mediate defense against toxic ROS, can reveal anti-microbial targets and shed light on pathogenic mechanisms. In this study, we provide evidence that a *Mycobacterium smegmatis* hemerythrin-like protein MSMEG_2415, designated MsmHr, is a H_2_O_2_-modulated repressor of the SigF-mediated response to H_2_O_2_. Circular dichroism and spectrophotometric analysis of MsmHr revealed properties characteristic of a typical hemerythrin-like protein. An *msmHr* knockout strain of *M. smegmatis* mc^2^155 (Δ*msmHr*) was more resistant to H_2_O_2_ than its parental strain, and overexpression of MsmHr increased mycobacterial susceptibility to H_2_O_2_. Mutagenesis studies revealed that the hemerythrin domain of MsmHr is required for the regulation of the H_2_O_2_ response observed in the overexpression study. We show that MsmHr inhibits the expression of SigF (MSMEG_1804), an alternative sigma factor that plays an important role in bacterial oxidative stress responses, including those elicited by H_2_O_2_, thus providing a mechanistic link between Δ*msmHr* and its enhanced resistance to H_2_O_2_. Together, these results strongly suggest that MsmHr is involved in the response of mycobacteria to H_2_O_2_ by negatively regulating a sigma factor, a function not previously described for hemerythrins.

## Introduction

Hydrogen peroxide (H_2_O_2_) is a universal oxidative stress molecule produced by aerobic organisms from all three domains of life (Imlay, 2008). The production of H_2_O_2_ by the host in response to pathogen infection is also an important innate defense mechanism (Fang, 2004). This ROS can be damaging via direct toxic effects or disruption of redox balance, the latter being critical for metabolic homeostasis and hence survival. An important intracellular pathogen, *Mycobacterium tuberculosis* has evolved many strategies to detoxify H_2_O_2_, some of which are unique to mycobacteria (Kumar et al., 2011; Trivedi et al., 2012). For example, *M. tuberculosis*, which lacks the conventional redox buffer glutathione, uses mycothiol (MSH), a low-molecular-weight thiol that exists in millimolar quantities in the cytoplasm, to generate a reducing environment (Farhana et al., 2010); and MSH-deficient mycobacterial mutants are hyper-susceptible to H_2_O_2_(Rawat et al., 2002). The precise mechanisms by which MSH protects against oxidative stress and redox imbalance remain to be determined. *M. tuberculosis* also lacks classic sensors such as OxyR and SoxR for the detection of redox signals (Imlay, 2013). The tubercle bacillus, however, expresses DosS and DosT (Kumar et al., 2007), two sensor histidine kinases whose heme iron plays a critical role in their response to the levels of O_2_, nitric oxide (NO) and carbon monoxide (CO). Interaction with these various gasses activates the kinase activity of DosS and DosT, relaying the signals to the response regulator DosR. WhiB3 is an Fe-S cluster transcription factor that controls the expression of the hypoxia regulon of *M. tuberculosis* (Bhat et al., 2012). Indeed, accumulating evidence suggests that the *M. tuberculosis* WhiB family of Fe-S cluster proteins plays important roles in regulating a wide spectrum of microbial functions including responses to oxidative stress and virulence (Burian et al., 2012; Saini et al., 2012a). Together, the DosS-DosT/WhiB3 paradigm underscores the importance of iron-containing proteins in the response of *M. tuberculosis* to redox signals, including those imposed by reactive oxygen intermediates.

The non-heme, di-iron, O_2_-binding hemerythrin-like proteins are present in all domains of life (Bailly et al., 2008; French et al., 2008). Bioinformatics analyses have revealed over 400 hemerythrin-like proteins in available prokaryotic genomes (Bailly et al., 2008; French et al., 2008). These proteins harbor the conserved hemerythrin domain either singly or jointly with another distinct functional domain (Bailly et al., 2008; French et al., 2008). Experimental evidence suggests that they can function as oxygen sensors and reserves, as well as mediate the delivery and transport of this diatomic gas (French et al., 2008). Dcr (*Desulfovibrio* chemoreceptor) H was the first bacterial hemerythrin-like protein to be identified. It has been proposed that the C-terminal hemerythrin domain of DcrH, a member of the Dcr family of putative methyl-accepting chemotaxis proteins of the anaerobic sulfate-reducing bacterium *Desulfovibrio vulgaris*, functions to sense O_2_ (Xiong et al., 2000); the signal thus generated is proposed to be transduced to the transmitting domains to mediate chemotaxis (Xiong et al., 2000). The first single-domain hemerythrin-like protein was identified in *Methylococcus capsulatus*. The expression of the *M. capsulatus* hemerythrin-like protein is enhanced significantly with increasing concentrations of copper, and is thought to be an oxygen carrier that supplies copper-containing methane monooxygenase with oxygen (Kao et al., 2004; Karlsen et al., 2005; Chen et al., 2012). Despite the wide distribution of hemerythrin-like proteins in a wide variety of bacterial species, including *M. tuberculosis* (Xiong et al., 2000; Karlsen et al., 2005; Isaza et al., 2006; Justino et al., 2007; Onoda et al., 2011; Schaller et al., 2012), functional characterization studies are scarce.

Transcriptional regulation is critical to bacterial survival in response to various stresses. Sigma factors are the primary transcriptional regulators of bacterial gene expression. *M. tuberculosis* has 13 sigma factors, and SigH, SigE, SigL, and SigF play important roles in ROS detoxification (Rodrigue et al., 2006). SigF, a highly conserved sigma factor in the genus *Mycobacterium* (Rodrigue et al., 2006), is highly induced by various environmental stresses and during stationary phase (Demaio et al., 1996). In *M. smegmatis, sigF* deletion also increases susceptibility to oxidative stress (Gebhard et al., 2008; Humpel et al., 2010). Recently, a genome-wide gene expression study (Humpel et al., 2010) and work from our laboratory (Wu et al., 2012) have provided evidence that SigF regulates the expression of oxidative stress defense genes such as *katA, dps1*, and *sodA*, but not *katG* and *ahpC*, genes that have been linked to mycobacterial resistance to isoniazid (Silva et al., 2003). These studies suggest that SigF-mediated resistance to H_2_O_2_ is independent of KatG and AhpC. This notion is also supported by the fact that SigF-deficient mutants do not display decreased susceptibility to isoniazid (Demaio et al., 1996; Humpel et al., 2010). Regulation of SigF in mycobacteria is generally thought to be predominantly at the post-transcriptional level via the action of anti-sigma and anti-anti-sigma factors (Michele et al., 1999; Beaucher et al., 2002; Singh and Singh, 2008). SigF is transcribed from two promoters, P*_msmeg_1802_* and P*_rbsw_* (Gebhard et al., 2008), and transcriptional reporter fusion studies have shown that promoter P*_msmeg_1802_* responds to entry into the stationary phase and promoter P*_rbsw_* is inducible upon treatment with D-cycloserine (Gebhard et al., 2008).

In this study, to investigate the role of hemorythrin-like proteins in mycobacteria, we cloned, expressed, and characterized the *M. smegmatis* mc^2^155 strain MsmHr protein (encoded by *msmeg2415*). Using a genetic and biochemical approach, we show that (i) MsmHr displays circular dichroism (CD) and UV-vis spectrophotometric features typical of a hemerythrin-like protein; (ii) relative to wild-type bacilli, the *msmHr* knockout strain (Δ*msmHr*) is more resistant to H_2_O_2_ and mc^2^155 overexpressing MsmHr exhibits enhanced H_2_O_2_ susceptibility; (iii) the H_2_O_2_ response is dependent on the hemerythrin domain; (iv) MsmHr represses *sigF* transcription through the promoter P*_rbsw_*, and thus participates in regulating the SigF-mediated H_2_O_2_ response. Our results indicate that MsmHr, the first mycobacterial hemorhythrin-like protein to be characterized, is involved in the H_2_O_2_ response in mycobacteria and provide insight into its mechanism.

## Materials and methods

### Culture medium and growth conditions

*M. smegmatis* cultures were grown in Middlebrook 7H9 medium (Becton Dickinson, Sparks, MD) supplemented with ADS enrichment (Albumin-Dextrose Saline containing 5% (w/v) Bovine serum albumin fraction V, 2% (w/v) D-Dextrose and 8.1% (w/v) NaCl) (Jacobs et al., 1991), 0.05% (v/v) Tween 80, and 0.5% (v/v) glycerol (Beijing Modern Eastern Finechemical Co. Ltd, Beijing). Middlebrook 7H10 medium supplemented with ADS enrichment and 0.5% (v/v) glycerol was used as the solid medium for examination of growth status. Growth was also examined in minimal Sauton's medium (4 g asparagine, 2 g sodium citrate, 0.5 g K_2_HPO_4_·3H_2_O, 0.5 g MgSO_4_·7H_2_O, 0.05 g ferric ammonium citrate, 60 g glycerol in 1 L of H_2_O supplemented with 0.05% (v/v) Tween 80) supplemented with antibiotics as indicated. Hygromycin (75 mg/L for *M. smegmatis*, 150 mg/L for *Escherichia coli*; Roche) and kanamycin (25 mg/L for *M. smegmatis*, 50 mg/L for *Escherichia coli*; Amresco) were added to the medium as needed. All bacterial strains used in this study are listed in Table S1.

### Generation of knockout mutant strains, complementation strains and overexpression strains

The *msmHr* (*msmeg_2415*) and *sigF* (*msmg_1804*) deletion mutants were generated via a specialized transducing phage delivery system as previously described (Bardarov et al., 2002). The 5'-flanking region of *msmHr* was amplified by polymerase chain reaction (PCR) with the 2415LL/2415LR primer pair and the 3'-flanking region of *msmHr* was amplified with the 2415RL/2415RR primer pair (all primers are listed in Supplemental Table S2. The flanking regions of *sigF* were generated by amplifying the upstream and downstream regions of *sigF* using the 1804LL/1804LR and 1804RL/1804RR primer pairs, respectively. Amplified fragments were ligated with plasmid p0004S, digested with *PflM*I (*msmHr*) or *Alwn*I (*sigF*), and allelic-exchange plasmids thus constructed were digested with PacI, and then ligated with PacI-digested phAE159. Phage packaging was performed using a MaxPlax packaging extract (Epicenter Biotechnologies, USA) to yield the knockout phages for *msmHr* (phAE-*msmHr*) and *sigF* (phAE-*sigF*). Specialized transduction was carried out as described previously (Bardarov et al., 2002). The knockout clones were screened by PCR using the primer pairs 2415InL/2415InR, 2415LLL/IL(R) and IR(F)/2415RRR for *msmHr* and 1804InL/1804InR, 1804LLL/IL(R), and IR(F)/1804RRR for *sigF*. Primer positions with respect to the appropriate genes are shown in **Figure 2A** and Figure S1. No *msmHr* or *sigF* mRNA was detected in the corresponding deletion strains by qRT-PCR using the appropriate primer pairs (**Figure 2A** and Figure S1). Complementation strains were constructed as described previously (Stover et al., 1991). Briefly, the full-length sequence of *msmHr* or *sigF* amplified from *M. smegmatis* genomic DNA was cloned into the integrating vector pMV361 (Stover et al., 1991) and the resultant plasmids were electroporated into the corresponding knockout strains to yield C-Δ*msmHr* (Δ*msmHr*::P*_hsp60_*-*msmHr*) and C-Δ*sigF* (Δ*sigF*::P*_hsp60_*-*sigF*). To over-express *msmHr*, the *msmHr* fragment was subcloned into pMV261 (Stover et al., 1991) to yield pMV261-*msmHr* for transformation into *M. smegmatis* (O-*msmHr*).

### Determination of survival phenotypes under stress

Early phase cultures (OD_600_ = 0.3) of all tested strains were serially diluted (1:10) and spotted (3 μl) onto solid 7H10 medium supplemented with ADS enrichment and stress-inducing chemical agents (20 μM streptonigrin, 250 μM NaNO_2_) or subjected to low-pH stress (7H9 supplemented with ADS enrichment and 0.05% Tween 80, pH 5.5). For peroxide stress, early-phase cultures were treated with 5 mM H_2_O_2_ for 3 h, serially diluted (1:10) and spotted (3 μl) onto solid 7H10 medium supplemented with 10% ADS. The optical density at 600 nm (OD_600_) was measured at the indicated times in the presence of various stresses. Survival under heat shock stress at 50°C was determined by the number of colony forming units during the time indicated. Statistical analyses were performed using unpaired two-tailed *t*-tests. *P*-values are only shown where significant differences were found. ^*^P < 0.05 and ^**^P < 0.01.

### Cloning, expression and purification of MsmHr in *Escherichia coli*

The coding sequence of *msmHr* was amplified from *M. smegmatis* mc^2^155 genomic DNA and cloned into the expression vector pET23b (+) (Novagen, USA), in-frame fused with a C-terminal 6xHis-tag sequence to generate the plasmid pET23b-*msmHr*, which was transformed into *E. coli* BL21 (AI) (Invitrogen, USA) for expression. Recombinant MsmHr was induced by incubation with 0.1% arabinose at 28°C for 3 h. Cells were harvested by centrifugation at 10,000 g for 5 min, resuspended in lysis buffer (20 mM Tris-HCl pH 8.0; 1 M NaCl, 10% (v/v) glycerol, 20 mM imidazole, 0.1% (v/v) Triton X-100, 1 mM phenylmethylsulfonyl fluoride (PMSF), 1 mg/ml lysozyme) and lysed by sonication. Lysates were centrifuged at 12,000 g for 30 min at 4°C to remove debris before purification. The supernatants were incubated with Ni-NTA agarose (Qiagen, USA) with rotation (15 g) for 4 h at 4°C. Beads were then washed three times with washing buffer (20 mM Tris-HCl pH 8.0, 0.5 M NaCl, 10% (v/v) glycerol, 50 mM imidazole, 0.1% (v/v) Triton X-100, 1 mM PMSF, 25 mM MgCl_2_). The proteins were eluted with elution buffer (50 mM Tris-HCl pH 7.5, 0.5 M NaCl, 25 mM MgCl_2_, 10% (v/v) glycerol, 400 mM imidazole) and protein concentration was measured using the bicinchoninic acid protein assay reagent and a bovine serum albumin standard. Purified protein was examined using 12% sodium dodecyl sulfate polyacrylaminde gel electrophoresis to verify molecular weight and purity (Figure S2).

### Spectrophotometric and circular dichroic (CD) analysis of MsmHr

Purified MsmHr was diluted in 20 mM Tris-Cl buffer (pH 7.5). Deoxy samples were obtained by adding a 10-fold molar excess of Na_2_S_2_O_4_ to MsmHr. UV-Vis spectrophotometric spectra were obtained in 1 mm path length quartz cuvettes on a UV-2802H UV-Vis spectrophotometer (Unico Shanghai Instruments Co., Ltd., China). Spectra of deoxy-MsmHr were collected in an anaerobic incubator (Shanghai Yuejin Medicial Instruments Co., Ltd, China). CD measurements were performed using a Chirascan Circular Dichroism Spectrometer (Applied Photophysics Ltd. UK). The analysis software provided with the instrument was used for analysis of the results.

### Determination of the minimum inhibitory concentration of isoniazid in *M. smegmatis*

The susceptibility of *M. smegmatis* to isoniazid (INH) was determined using the broth microdilution method (Wallace et al., 1986). After two-fold dilutions of INH in 7H9 supplemented with ADS enrichment and 0.5% (v/v) glycerol, 40-μl aliquots were mixed with 40 μl of *M. smegmatis* suspension (10^5^ cells/ml) and deposited into wells of 96-well microtiter plates. The highest concentration of INH was 100 μg/ml. Plates were incubated at 37°C for 2 days and OD_600_ values of cultures were then measured using a microplate reader (FLUOstar OPTIMA, BMG Labtech). The minimum inhibitory concentration (MIC) was defined as the lowest concentration of drug that inhibited the visible bacterial growth of *M. smegmatis* after a 2-day incubation (OD_600_ < 0.05). INH susceptibility tests were repeated at least 3 times.

### RNA isolation, RT-PCR and quantitative PCR

Log phase cultures (OD_600_ = 0.8–1.0) of all tested strains were diluted 1:100 in 7H9 media supplemented with ADS enrichment, 0.5% (v/v) glycerol and 0.05% (v/v) Tween 80. Strains were cultured until the OD_600_ reached 0.3 and then divided into control and treatment groups. In the treatment group, the cells were treated with 5 mM H_2_O_2_ for 30 min, and then collected by centrifugation at 12,000 g. Bacterial pellets were resuspended in TRIzol (Invitrogen, USA) and RNA was purified according to the manufacturer's instructions. cDNA was synthesized using the SuperScriptTM III First-Strand Synthesis System (Invitrogen, USA). Quantitative real-time PCR (qRT-PCR) was performed in a Bio-Rad iCycler using 2x SYBR real-time PCR pre-mix (Takara Biotechnology Inc., Japan). The following cycling program was used: 95°C for 1.5 min followed by 40 cycles of 95°C for 10 s, 60°C for 15 s, and 72°C for 15 s, followed for 72°C for 6 min. *M. smemgatis rpoD* encoding RNA polymerase sigma factor SigA was selected as a reference to normalize gene expression. The 2^−ΔΔCT^ method was used (Livak and Schmittgen, 2001) to evaluate the relative gene expression in different strains and/or different treatments. Primers used are listed in Table S2.

### Construction of promoter-*lacZ* fusion expression vectors

SigF is reported to be transcribed from two promoters, P*_msmeg_1802_* and P*_rbsw_* (Gebhard et al., 2008). To construct the P*_msmeg_1802_*-*lacZ* and P_rbsW_-*lacZ* plasmids, we used the reported primer pairs PsigFF/PsigFR and P1802F/P1802R (Gebhard et al., 2008), and P*_msmHr_*-lacZ using P2415F/P2415R (Supplemental Table S2). The promoter sequences of *sigF, msmeg_1802* and *msmHr* were then cloned into the upstream region of the *lacZ* gene in pLACZint (Vasudeva-Rao and McDonough, 2008), generating the plasmids P_rbsW_-*lacZ*, P*_msmeg_1802_*-*lacZ* and P*_msmHr−_lacZ*, respectively.

### β-galactosidase (*lacZ*) activity assay

The β-galactosidase activity of various strains were determined as described previously (Gebhard et al., 2008). Briefly, cultures were collected at early logarithmic phase (OD_600_ ≈ 0.3) and resuspended in Z buffer (60 mM Na_2_HPO_4_, 40 mM NaH_2_PO_4_, 10 mM KCl, 1 mM MgSO_4_ and 50 mM β-mercaptoethanol). Cells were then lysed using a Fastprep bead-beater (Biospec). The enzyme reaction was initiated by adding ortho-nitrophenyl-β-D-galactopyranoside (2 mg/ml) and terminated by adding Na_2_CO_3_(0.8 M). β-galactosidase activity was assessed by measuring the OD_420_ value of the reactions mixtures. Assays were performed in triplicate, and the Miller units (MU) were calculated as follows: 1000 × OD_420_/(OD_600_ of assayed culture × assayed volume × time).

### Construction of msmHr point mutants

Mutation of specific amino acids was incorporated into the hemerythrin-like domain of *msmHr* in pMV261-*msmHr* by mismatched PCR primers (Table S2). Site-directed mutagenesis was performed following using a QuikChange® Site-Directed Mutagenesis Kit (Stratagene) according to the manufacturer's instructions. Mutant clones were confirmed by DNA sequencing (BGI-Shenzhen, Shenzhen China). pMV261-*msmHr* mutants were transformed into *M. smegmatis* mc^2^155. Mutated sites are as follows (altered amino acids are in underlined bold font):

H1M: ^55^AV**H**ETA**EE**MV^65^ → ^55^AV**L**ETA**AA**MV^65^
H2M: ^86^EE**H**KAK**QQ**LS^96^ → ^86^EE**L**KAK**AA**LS^96^
H3M: ^122^AA**H**EEA**EE**FV^132^ → ^122^AA**L**EEA**AA**FV^132^

## Results

### MsmHr is a bacterial hemerythrin-like protein

Detoxification strategies for scavenging host immune defense system-derived H_2_O_2_ are important for the intracellular survival of mycobacterial pathogens. Expression of Fe-related proteins, such as the Fe-S cluster transcription factor WhiB3, and heme-iron sensors DosS and DosT, is an important strategy for regulating redox balance (Bhat et al., 2012). Hemerythrin proteins are iron-binding proteins known to be involved in oxygen transport and storage, but their biological functions in mycobacteria have yet to be elucidated. Three proteins in *M. smegmatis*, MsmHr, Msmeg_3312 and Msmeg_6612, are predicted to be hemerythrin-like proteins, however, as preliminary experiments indicated that only MsmHr is related to the H_2_O_2_ response (data not shown), we focused our attention on MsmHr.

**Figure 1:**
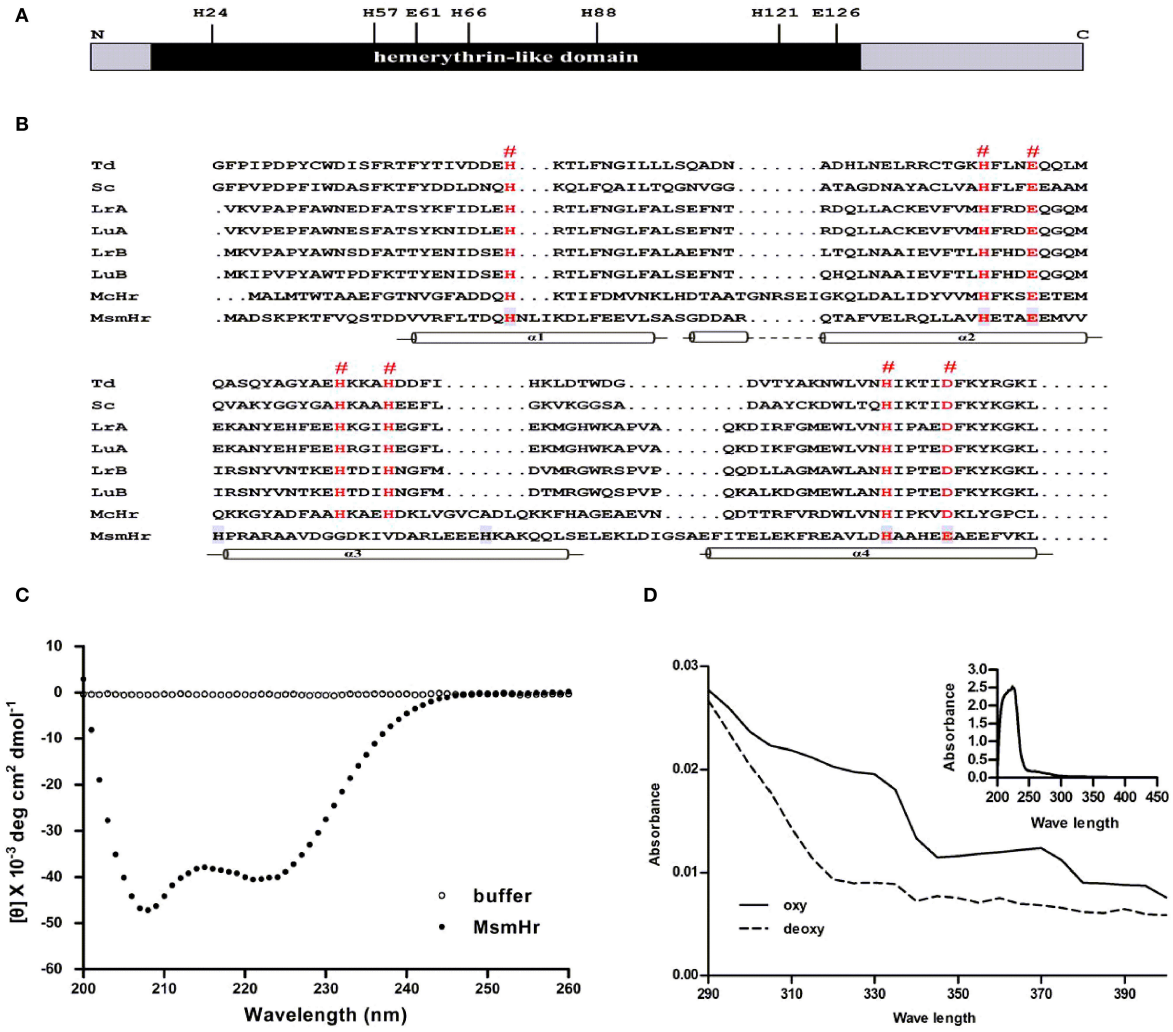
**MsmHr is a hemerythrin-like protein**. **(A)** Conserved residues of hemerythrin-like domain in MsmHr. H: histidine; E: glutamate. **(B)** MsmHr sequence analysis. Alignment of the hemerythrin-like domain of MsmHr with other hemerythrin family proteins. Hemerythrin sequences compared are from *Themiste dyscrita* (Td), *Siphonosoma cumanense* (Sc), *Lingula reevii* (α chain and β chain; LrA and LrB), and *Lingula unguis* (α chain and β chain; LuA and LuB), *Methylococcus capsulatus* (McHr). The predicted Fe^2+^ binding sites (^#^) are indicated in red font. Helices (α1, α2, α3, and α4) are indicated below the sequences. **(C)** CD spectral analysis of MsmHr. Blank control: 20 mM Tris-Cl buffer, pH 7.5 alone (clear circle); MsmHr: 100 mg/L MsmHr in 20 mM Tris-Cl buffer, pH 7.5 (black circle). Measurements were obtained at room temperature. Images are representative of 5 independent experiments. **(D)** UV/Vis spectral analysis of MsmHr. Reduced (deoxy) and oxidized (oxy) MsmHr. Inset: representative spectra for 100 mg/L MsmHr. Images shown are representative of 3 independent experiments.

We first constructed a multiple alignment of MsmHr with other hemerythrin-like proteins from different bacterial species (Figure 1A). Residues H24, H57, E61, H66, H88, H121, and E126 (numbering based on the MsmHr sequence) matched the characteristic motifs H… HxxxE … HxxxH… HxxxxD/E of hemerythrin domains. The secondary structure predicted by SWISS-MODEL (http://swissmodel.expasy.org/) (Guex and Peitsch, 1997; Schwede et al., 2003; Arnold et al., 2006; Kiefer et al., 2009) suggested that MsmHr has a typical hemerythrin structure with four α-helices (residues 16–40, 41–68, 80–103, and 104–132) (Figure 1B).

To confirm the predicted helical structure of MsmHr, we purified MsmHr-His_6_protein from *E. coli*. Circular dichroism (CD) spectra of the *E. coli*-purified MsmHr showed two minima at 208 and 222 nm (Figure 1C). Such a pattern, which is characteristic of protein α-helical structures, is typical of the circular dichroism spectra of previously analyzed bacterial hemerythrins (Wirstam et al., 2003). We also performed a CD analysis of gelactin AAL (*Agrocybe aegerita* lectin, PDB 2ZGU), a protein which consists mainly of β-sheets. The CD spectra showed only one minima at 210 nm (Supplemental Figure S3). The UV-visible absorption spectra of MsmHr showed peaks at 327 and 376 nm, a pattern ascribable to the di-iron-center of hemerythrin-like proteins (Karlsen et al., 2005). Absorbance peaks were abrogated upon reduction with Na_2_S_2_O_4_ to generate the deoxy form by removing oxygen (Figure 1D). Since MsmHr has no Trp and only one Phe, UV/Vis spectrophotometric analysis of the purified protein did not detect absorbance at 280 nm (Figure 1D inset). Taken together, these bioinformatics, CD and spectrophotometric results strongly suggest that MsmHr is a hemerythrin-like protein.

### MsmHr is involved in the H_2_O_2_ stress response

**Figure 2:**
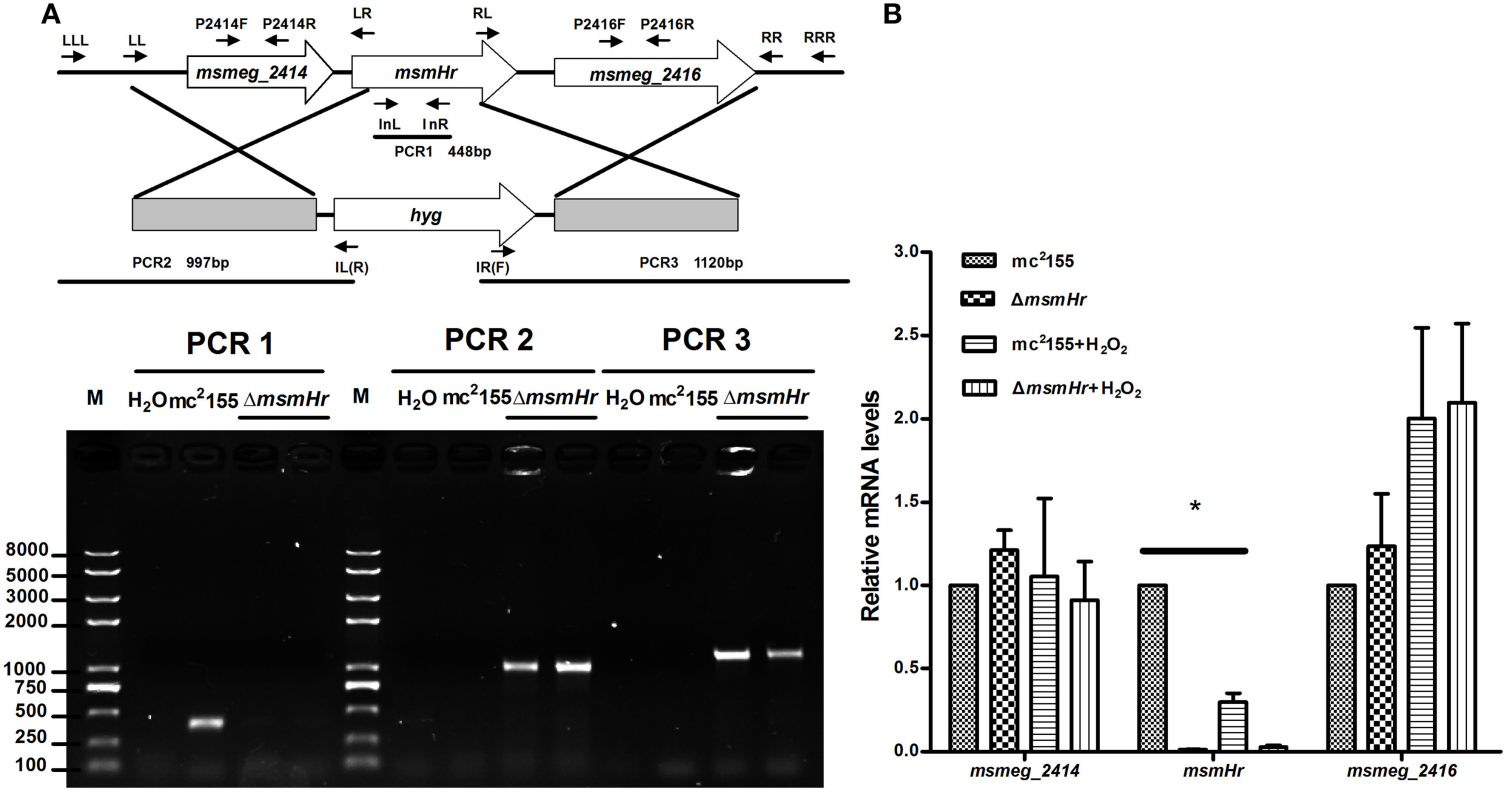
**Generation of the Δ*msmHr M. smegmatis* strain**. **(A)** Genomic organization of the *msmHr* gene locus. (Upper panel) Genes are shown as large arrows in their native orientation. Small arrows represent the forward and reverse primers used for PCR, and sizes of the amplified products are indicated. Location and orientation of the hygromycin cassette are also indicated (Bottom panel). No PCR product was obtained using primers 2415InL and 2415InR to amplify the coding sequences of *msmHr*. PCR products for the upstream and downstream regions of *msmHr* were amplified using the primer pairs 2415LLL/IL(R) and 2415RRR/IR(F), respectively. **(B)** Effect of *msmHr* deletion on *msmeg_2414* and *msmeg_2416* expression. Quantitative real-time PCR (qRT-PCR) analysis of *msmHr* cluster transcription. The primer pairs 2414qF/2414qR and 2416qF/2416qR were used for qRT-PCR. Results are shown as the means ± standard deviations of three replicates (^*^P < 0.05).

To define the biological functions of MsmHr, a deletion mutant, Δ*msmHr*, was generated by specialized transduction (Bardarov et al., 2002) (Figure 2A). The loss of *msmHr* was confirmed by PCR (Figure 2A) and no *msmHr* mRNA was detected in Δ*msmHr* (Figure 2B). To determine if deletion of *msmHr* had polar effects on the *msmeg_2414* and *msmeg_2416* genes, their mRNA levels were compared in the wild type mc^2^155 and Δ*msmHr* strains by RT-PCR. Statistically significant differences in *msmeg_2414* and *msmeg_2416* mRNA were not detected (Figure 2B), indicating that *msmHr* knockout did not affect the transcriptional levels of *msmeg_2414* and *msmeg_2416*.

**Figure 3:**
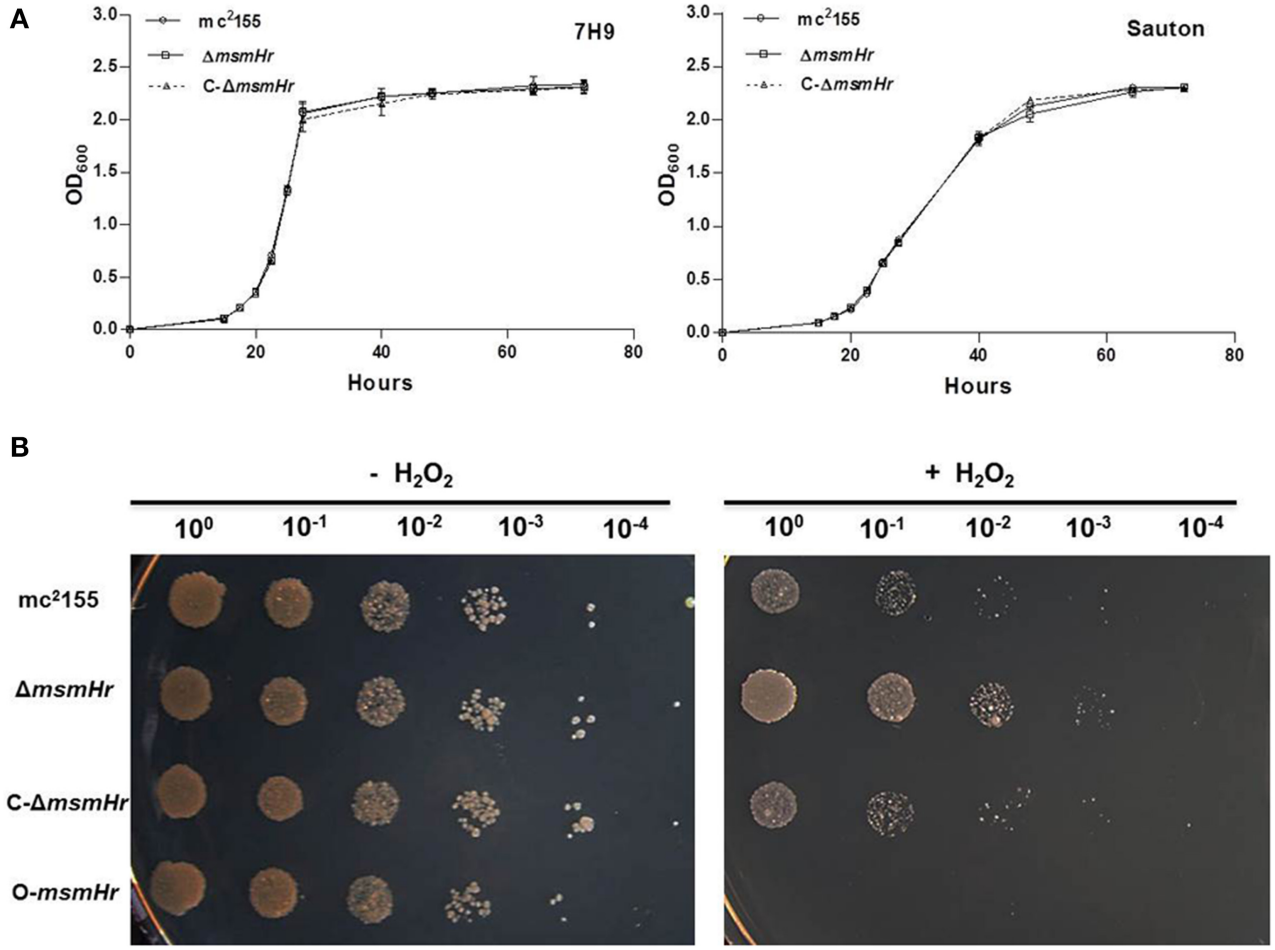
**MsmHr is involved in the H_2_O_2_ stress response**. **(A)** MsmHr is not necessary for normal growth. Growth curves of mc^2^155, Δ*msmHr* and the complementary strain (C-Δ*msmHr*) in 7H9 rich media (left) and Sauton minimal media (right). The data shown are representative of four independent experiments. **(B)** MsmHr plays a negative role in the bacterial H_2_O_2_ response. The left panel represents serial dilutions (1:10) of mc^2^155, Δ*msmHr*, the complemented strain C-Δ*msmHr* and the *msmHr* overexpression strain (O-*msmHr*: pMV261-*msmHr*/mc^2^155). Diluted log phase *M. smegmatis* cultures were spotted (3 μl) onto solid 7H10 medium supplemented with 10% ADS. Right panel, the corresponding strains were spotted on solid 7H10 medium supplemented with 10% ADS after treatment with 5 mM H_2_O_2_. Photographs were taken after 2–3 days incubation at 37°C. Images shown are representative of at least 3 experiments.

We then compared the growth rates of the *M. smegmatis* parental strain mc^2^155 with the MsmHr-deficient mutant Δ*msmHr* in both 7H9 rich medium and Sauton's minimal medium. No growth abnormalities were detected in Δ*msmHr* relative to mc^2^155, demonstrating that MsmHr does not influence *M. smegmatis* growth in rich (7H9) or minimal medium (Sauton) (Figure 3A). To identify possible biological roles of MsmHr, the growth kinetics and survival of Δ*msmHr* were examined under various stress conditions, including NO, hypoxia, H_2_O_2_, heat shock, and acidic pH. No growth defects in Δ*msmHr* were detected under the conditions tested, with the exception of H_2_O_2_ stress (Figure 3B). Δ*msmHr* exhibited mild H_2_O_2_ resistance compared to the wild type mc^2^155 strain after treatment with 5 mM H_2_O_2_ for 3 h, while no difference in the growth of the two strains was observed under non-H_2_O_2_ treatment conditions. In addition, the resistance phenotype was abrogated in the complemented strain, C-Δ*msmHr* (Figure 3B). Moreover, overexpression of *msmHr* in mc^2^155 (O-*msmHr*) was associated with H_2_O_2_ susceptibility after treatment with 5 mM H_2_O_2_ for 3 h (Figure 3B). Differences in mRNA levels of *msmeg_2414* and *msmeg_2416* in wild type mc^2^155 and Δ*msmHr* were not detected after treatment with H_2_O_2_(Figure 2B). A statistically significant reduction in *msmHr* mRNA levels was found between mc^2^155 in the presence and absence of H_2_O_2_ (Figure 2B). Taken together, our data suggest that *msmHr* plays an inhibitory role in the response to H_2_O_2_ stress.

### The hemerythrin-like domains of MsmHr are required for the response to H_2_O_2_ stress

**Figure 4:**
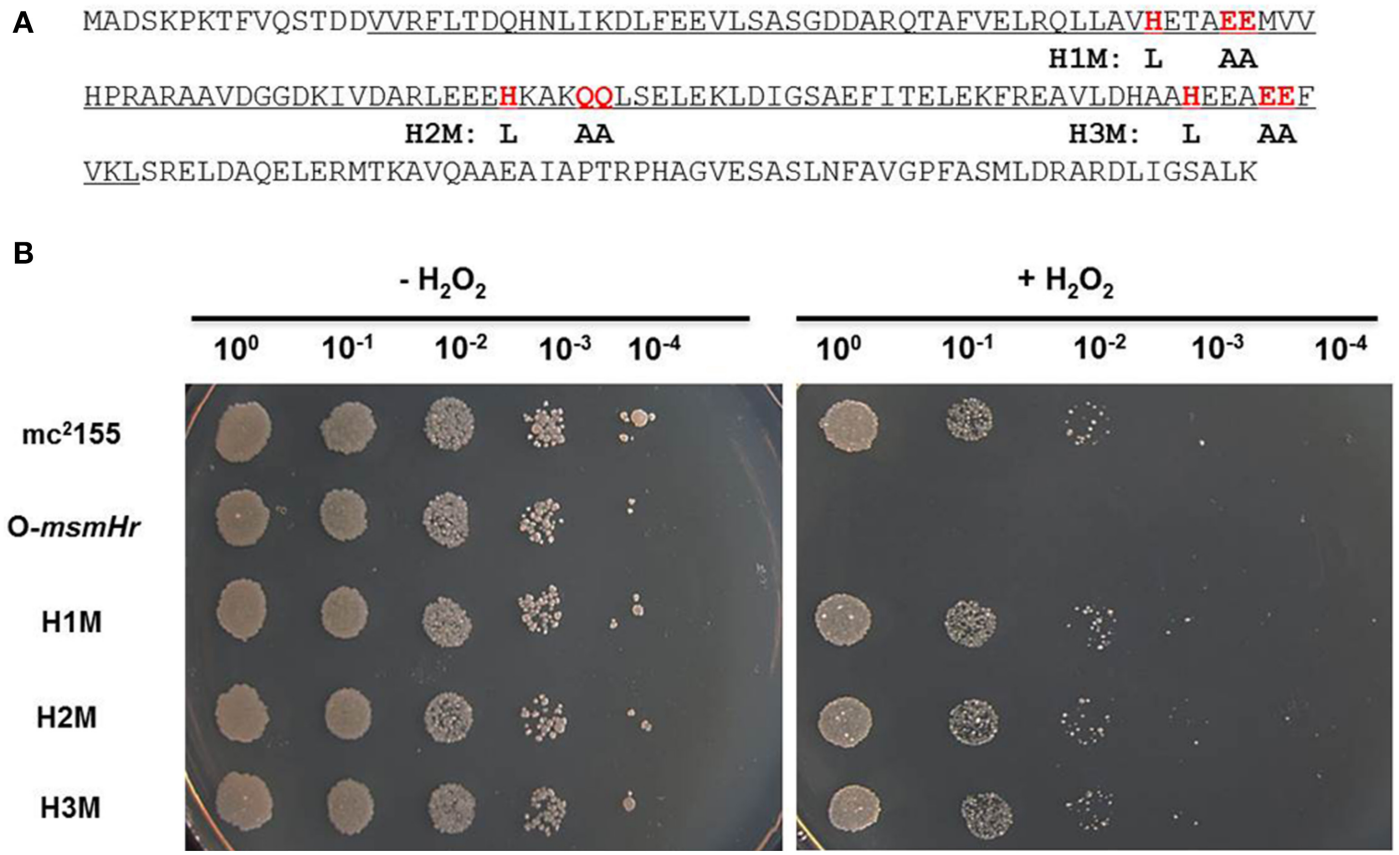
**The hemerythrin domain is essential to MsmHr function**. **(A)** The hemerythrin domain (underlined) of MsmHr and conserved amino acids (H… EE/QQ: red and boxed) in the hemerythrin domain are indicated. Mutations used in the following experiments are indicated under the sequence. **(B)** Serial dilutions (1:10) of mc^2^155, pMV261-*msmHr*/mc^2^155 (O-*msmHr*), pMV261-*msmHr*H1M/mc^2^155 (H1M), pMV261-*msmHr*H2M/mc^2^155 (H2M) and pMV261-*msmHr*H3M/mc^2^155 (H3M) treated with 0 (left) or 5 mM (right) H_2_O_2_ were spotted (3 μl) onto solid 7H10 agar. Photographs were taken after 2–3 days incubation at 37°C. Images shown are representative of at least 3 experiments.

To evaluate the potential roles of the hemerythrin-like domain of MsmHr in the H_2_O_2_ response, we constructed 3 mutants, H1M, H2M, and H3M, in which the respective conserved amino acid motifs HxxxEE, HxxxQQ, and HxxxEE were all mutated to LxxxAA (Figure 4A). As overexpression of *msmHr* showed higher susceptibility to H_2_O_2_, overexpression vector pMV261 containing *msmHr* alleles harboring the point mutations H1M, H2M or H3M was introduced into mc^2^155 which was then assessed for resistance to H_2_O_2_. All strains overexpressing the corresponding H1M, H2M or H3M mutant *msmHr* proteins behaved like mc^2^155. By contrast, strains containing intact *msmHr* proteins were susceptible to H_2_O_2_ (Figure 4B). This result indicates that the hemerythrin-like domain of MsmHr is required for H_2_O_2_ susceptibility.

### MsmHr represses *sig*F expression through the promoter P_*rbsw*_

**Figure 5:**
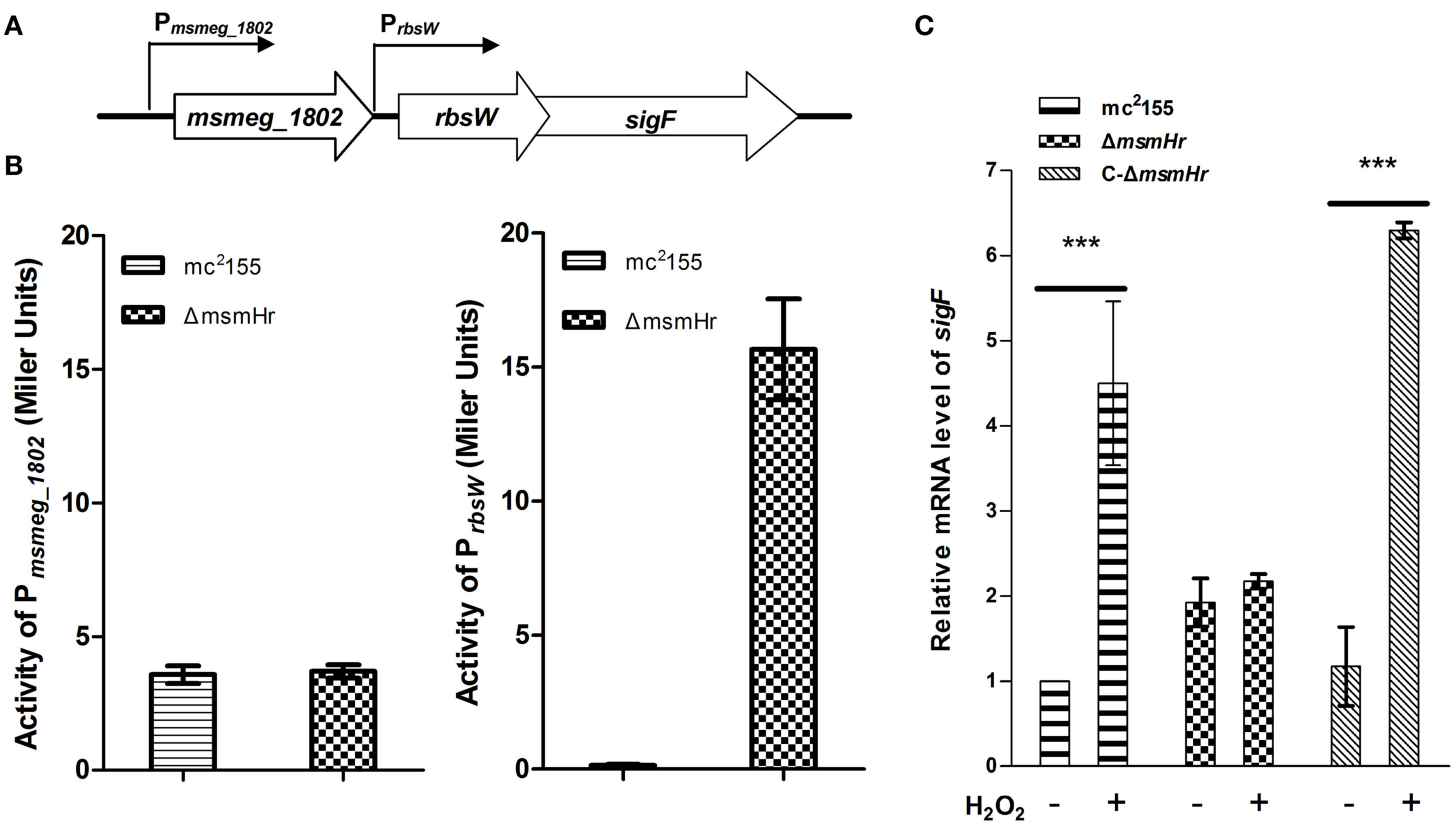
**MsmHr regulates *sigF* expression through the promoter P*_rbsw_***. **(A)** The genomic organization of the *sigF* locus. Genes are shown as large arrows in their transcriptional orientation, and the locations of P*_msmeg_1802_* and P*_rbsw_* are indicated. **(B)** MsmHr negatively regulates the activity of the *sigF* promoter P*_rbsw_*. P*_msmeg_1802_*-*lacZ* and P*_rbsw_*-*lacZ* were each transformed into mc^2^155 and the mutant strain Δ*msmHr*. The β-galactosidase activity was measured when bacterial growth reached an OD_600_ of 0.3. Results representative of 5 independent experiments are shown. **(C)** The *msmHr* deletion is associated with an increase in *sigF* mRNA levels. Early-phase cultures of *M. smegmatis* mc^2^155, Δ*msmHr* and the complemented strain C-Δ*msmHr* were treated with 0 (−) or 5 (+) mM H_2_O_2_ for 30 min, and *sigF* expression levels were determined by qRT-PCR. Results are shown as the mean ± standard deviations of three replicates (^***^P < 0.01).

There are two independent oxidative stress pathways in mycobacteria: the KatG-, isoniazid (INH)-related pathway and the SigF-related, INH-unrelated related pathway (Gebhard et al., 2008; Wu et al., 2012). INH is an important first-line anti-mycobacterial pro-drug and is activated by the bacterial catalase-peroxide enzyme encoded by *katG* (*msmeg_3461*). Resistance to H_2_O_2_ has been shown to correlate with susceptibility to INH (Bulatovic et al., 2002). To determine which pathway MsmHr is involved in, we measured the minimum inhibitory concentration (MIC) of INH against Δ*msmHr* and wild type mc^2^155. No difference in the MICs of these two strains was detected (3.125 mg/L in both Δ*msmHr* and mc^2^155), suggesting that MsmHr is not involved in the INH-related H_2_O_2_ response. We then examined whether MsmHr is involved in the SigF-mediated H_2_O_2_ response. As previous studies have shown that *sigF* is transcribed from two promoters, we constructed two vectors with the two promoter regions, P*_msmeg_1802_* and P*_rbsw_*, fused to *lacZ* (P*_msmeg_1802_*-*lacZ* and P*_rbsw_*-*lacZ*, respectively) (Humpel et al., 2010). We measured the indicated promoter activities at the early logarithmic phase: β –galactosidase activity associated with P*_msmHr_*-*lacZ* was 6.9 ± 0.2 MU in mc^2^155 and 7.6 ± 0.1 MU in Δ*msmHr*, indicating that MsmHr does not self-regulate at the transcriptional level. Wild type mc^2^155 harboring P*_msmeg_1802_*–*lacZ* had a β –galactosidase activity of 3.6 ± 0.3 MU, while the activity of Δ*msmHr* harboring P*_msmeg_1802_*–*lacZ* was 3.7 ± 0.2 MU, indicating that MsmHr does not influence the promoter activity of P*_msmeg_1802_* (Figure 5B left panel). In contrast, Δ*msmHr* harboring P*_rbsw_*-*lacZ* had a significantly higher β –galactosidase activity (15.7 ± 1.9 MU) than that of wild type mc^2^155 (Figure 5B right panel). We also measured the mRNA levels of *sigF* in mc^2^155, Δ*msmHr* and its complementary strain C-Δ*msmHr*. Consistent with results for the promoter, the knockout *msmHr* led to a 1.9 ± 0.2 fold increase relative to the mRNA level of *sigF* to wild type mc^2^155, while levels of *sigF* mRNA were not significantly different (Figure 5C). Taken together, our results suggest that MsmHr affects the mRNA level of *sigF* via P*_rbsw_*.

### MsmHr is necessary for the SigF mediated H_2_O_2_ response

The above results indicate that MsmHr suppresses *sigF* expression at the early logarithmic phase (Figure 5). We next examined the influence of MsmHr on the SigF-mediated H_2_O_2_ pathway. We compared the mRNA level of *sigF* between mc^2^155, Δ*msmHr* and the Δ*msmHr* complementary strain C-Δ*msmHr* after 5 mM H_2_O_2_ treatment for 30 min. As shown in Figure 5C, *sigF* mRNA was induced by H_2_O_2_ in mc^2^155, while induction of *sigF* mRNA by H_2_O_2_ was not detected in Δ*msmHr*. The increase in *sigF* mRNA induced by H_2_O_2_ was restored in C-Δ*msmHr*. These results show that MsmHr is required for the SigF-mediated response to H_2_O_2_.

**Figure 6:**
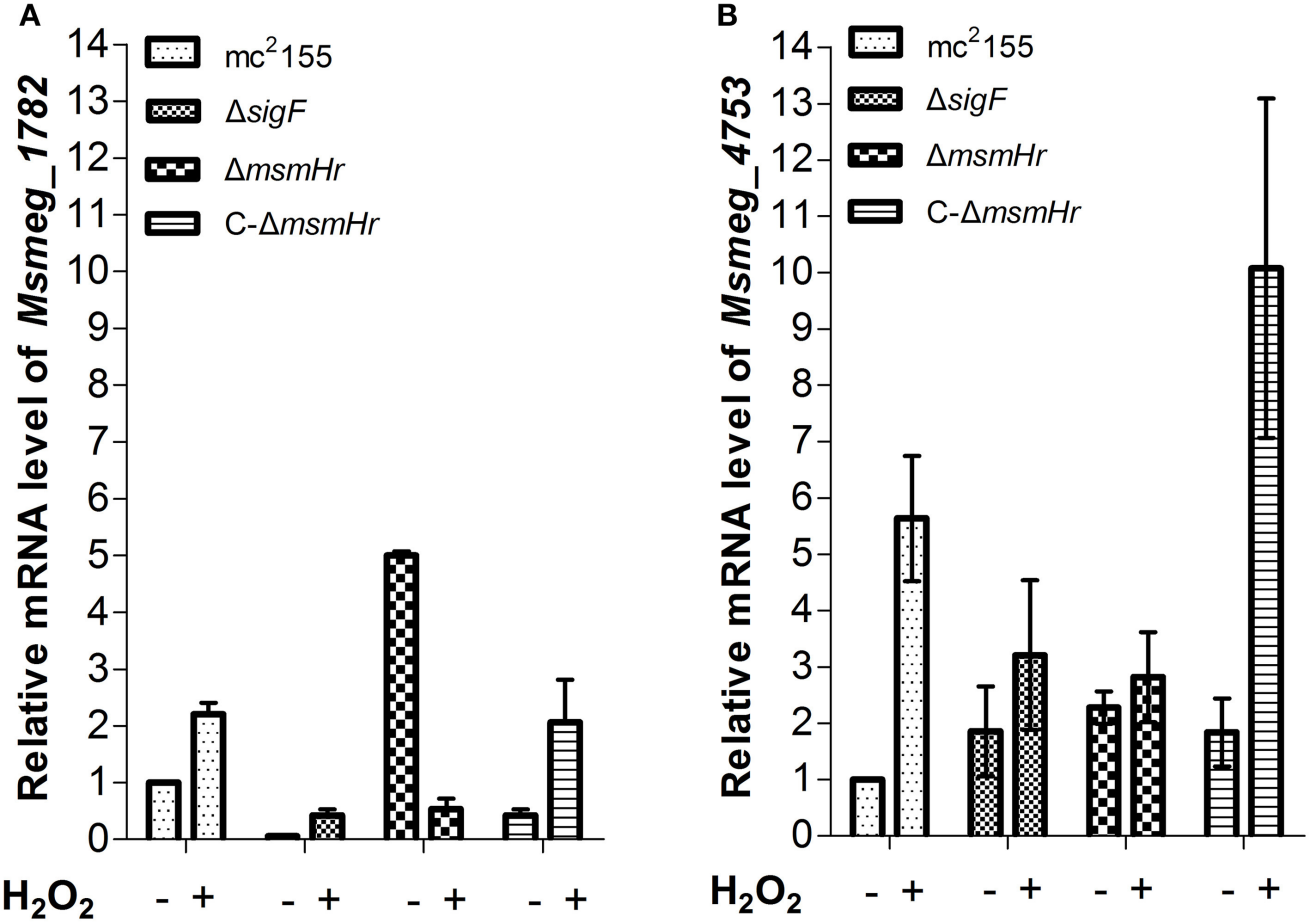
**(A)** Corresponding mRNA level of *msmeg_1782* was determined by qRT-PCR. **(B)** Corresponding mRNA level of *msmeg_4753* was determined by qRT-PCR. Bacteria were treated as described in Figure 5. Data shown are representative of three independent experiments.

In addition, we used qRT-PCR to measure the mRNA levels of redox-related genes in both mc^2^155 and Δ*msmHr* in response to H_2_O_2_ treatment, using *rpoD* mRNA as an internal invariant control (Table S3). We then chose the high H_2_O_2_-induced genes *msmeg_4753* and *msmeg_1782*, which belong to the SigF regulon, to evaluate their mRNA level in response to H_2_O_2_ treatment in mc^2^155, Δ*sigF*, Δ*msmHr and* C-Δ*msmHr*. The level of *msmeg_4753* mRNA increased 5.6 ± 0.9 fold in mc^2^155 after treatment with H_2_O_2_, but induction of *msmeg_4753* was abrogated in Δ*msmHr* and Δ*sigF* after treatment with H_2_O_2_ (Figure 6B). In C-Δ*msmHr*, an increase in *msmeg_4753* RNA was observed in response to H_2_O_2_ (Figure 6B). The level of *Msmeg_1782* mRNA increased two-fold in both mc^2^155 and C-Δ*msmHr* when treated with H_2_O_2_, while no changes in mRNA level were observed in response to H_2_O_2_ in Δ*msmHr* and Δ*sigF* (Figure 6A). Taken together, this data indicates that MsmHr is required for the SigF-mediated H_2_O_2_ response.

## Discussion

In this study, we have identified a mycobacterial hemerythrin-like protein MsmHr, which regulates *sigF* expression via promoter P*_rbsw_* and is necessary for the SigF-mediated H_2_O_2_ response. To our knowledge, MsmHr is the first hemerythrin-like protein to be characterized in mycobacteria.

Specific roles for hemerythrin-like proteins are just beginning to be characterized (Xiong et al., 2000; Justino et al., 2007; Schaller et al., 2012). On the basis of their sequences, hemerythrin-like proteins have been postulated to have diverse physiological functions related to oxygen and/or iron (Bailly et al., 2008; French et al., 2008). For example, the *E. coli* hemerythrin-like protein YtfE confers protection against both NO and H_2_O_2_ stresses (Justino et al., 2005, 2007). Here, however, we did not observe any growth differences between mc^2^155 and Δ*msmHr* under NO stress, suggesting that the functions of hemerythrin-like proteins in mycobacteria might be distinct from those in *E. coli*. The variation in biological functions of hemerythrin-like proteins might be due to different selective evolutionary environments (Saini et al., 2012b; Martin-Duran et al., 2013).

No difference in sensitivity to INH was observed between the wild-type mc^2^155 and Δ*msmHr* strains, suggesting that MsmHr is not involved in the INH-related oxidative stress response pathway but rather in an alternative SigF-related H_2_O_2_ pathway (Gebhard et al., 2008; Wu et al., 2012). It will be interesting to explore why mycobacteria use two-independent H_2_O_2_ scavenging pathways and which sensors trigger each of these signaling pathways. Recent reports show that a hemerythrin-like domain of FBXL5 can sense if endogenous iron is limiting and respond to iron stress in the mammalian system (Salahudeen et al., 2009; Vashisht et al., 2009). The correlation between iron- and oxygen- binding MsmHr and SigF-dependent H_2_O_2_ responses needs to be further explored.

The complexity of the transcription regulatory network allows for efficient and prompt change in levels of gene transcription in response to environmental changes. The protein encoded by mycobacterial *sigF* has closest homology to *Streptomyces coelicolor* SigF, *Bacillus subtilis* SigF and *B. subtilis* SigB (Demaio et al., 1996, 1997; Gebhard et al., 2008). In *B. subtilis, sigB* is activated upon entry into the stationary phase and by environmental stresses such as heat, oxidative stress and hyper osmosis. The transcription of sigB has been shown to be controlled by two promoters (Wise and Price, 1995). Similarly, SigF may be a potential general stress regulator; SigF is not only activated upon entry into the stationary phase, but is also induced by environmental stresses such as heat shock, acidic pH and oxidative stress (Wise and Price, 1995; Gebhard et al., 2008). Expression of *sigF* has been shown to be regulated by two promoters, P*_msmeg_1802_* and P*_rbsw_*. While promoter P*_msmeg_1802_* is known to respond to entry into the stationary phase (Gebhard et al., 2008), the role of P*_rbsw_* is less well understood. In this study, comparisons of the activity of the promoters of *sigF* and the mRNA level of *sigF* in the wild type mc^2^155 and mutant Δ*msmHr* strains (Figure 5) showed that MsmHr regulates *sigF* expression via the P*_rbsw_* promoter.

We show here that MsmHr is essential for the SigF-mediated H_2_O_2_ response (Figures 5, 6). MsmHr hinders *sigF* promoter activation and inhibits *sigF* transcription during normal growth. When *msmHr* is deleted, inhibition of *sigF* is abrogated, and *sigF* maintains a higher transcript level. The transcription level of *sigF* did not vary in response to H_2_O_2_ treatment in mutant Δ*msmHr* strains, possibly because the mRNA level of *sigF* was maintained at a higher level in Δ*msmHr* (Figure 5). We measured mRNA level changes of members of the SigF-regulon, *msmeg_4753* and *msmeg_1782*, before, between, and after treatment with H_2_O_2_. Our data show that in wild type mc^2^155, mRNA levels of both *msmeg*_*4753* and *msmeg*_*1782* increased in response to H_2_O_2_treatment, but no specific response to H_2_O_2_ was observed in Δ*sigF* and Δ*msmHr* (Figure 6), suggesting that MsmHr is required for induction of *msmeg*_*4753* and *msmeg*_*1782* transcription in response to H_2_O_2_. The presence of inhibitory protein MsmHr suggests that the transcriptional regulation of SigF itself and the SigF regulon, or at least part of the SigF regulon, is based on the balance between the activation and inhibition of the H_2_O_2_ response. In Δ*msmHr*, the inhibition of *sigF* is abrogated and *sigF* maintains high transcript levels. The expression of one of the SigF regulon genes, *msmeg_1782*, is high in Δ*msmHr* during normal growth compared with mc^2^155, but decreases in response to H_2_O_2_ treatment. This is consistent with the finding that MsmHr is necessary for the SigF-dependent H_2_O_2_ pathway and that the response of *sigF* to H_2_O_2_ is also abrogated in the absence of *msmHr*.

In summary, we have identified a mycobacterial hemerythrin-like protein that negatively regulates SigF via the P*_rbsw_* promoter in response to oxidative stress.

### Conflict of interest statement

The authors declare that the research was conducted in the absence of any commercial or financial relationships that could be construed as a potential conflict of interest.
