# Supplementary material for: A bacterial hemerythrin-like protein MsmHr inhibits the SigF-dependent hydrogen peroxide response in mycobacteria
Source: Front Microbiol. 2015 Jan 15;5:800. doi: 10.3389/fmicb.2014.00800 (PMC4295536; doi:10.3389/fmicb.2014.00800)
Supplement: Supplementary file 1 [file Table1.DOC]

**Table S1. Bacterial strains and plasmids used in this study**

| Name | Relevant genotype or description | Reference |
| --- | --- | --- |
| **Strains** |  |  |
| *Mycobacterium smegmatis* |  |  |
| mc^2^155 | Wild type | W.R. Jacobs |
| Δ*msmHr* | Deletion mutant of *msmeg_2415* (*msmHr*) | This work |
| Δ*sigF* | Deletion mutant of *msmeg_1804* (*sigF*) | This work |
| C-Δ*msmHr* | Δ*msmHr* containing pMV361-*msmHr* which contains full length *msmHr* | This work |
| O-*msmHr* | mc^2^155 containing pMV261-*msmHr* which contains full length *msmHr* | This work |
| *E. coli* |  |  |
| DH5α | F− *recA1* *hsdR17* *thi*-*1* *gyrA96* *supE44* *endA1relA1* *recA1* *deoR* Δ(*lac*Z*YA*-*argF*)U169 (*φ*80*lac*ZΔM15) |  |
| BL21(AI) | Chromosome contains T7 RNA polymerase gene (T7 RNAP) | Invitrogen |
| **Plasmids** |  |  |
| pMV261 | *Mycobacterial* extrachromosomal expression vector, Km^R^ |  |
| pMV361 | *Mycobacterial* integrative expression vector, Km^R^ |  |
| phAE159 | Temperature-sensitive mycobacteriophage phasmid, Amp^R^ |  |
| p0004S | Clone vector for construction allelic exchange substrate plasmid, Hyg^R^ | (2) |
| pET23b(+) | Expression vector carrying an N-terminal T7•Tag sequence plus an optional C-terminal His•Tag  Sequence, Amp^R^ | Novagen |
| pMV361-*msmHr* | pMV361 containing full sequence of *msmHr*, Km^R^ | This work |
| phAE159-*msmHr* | phAE159 containing ~700bp fragments up- and ~900bp downstream of *msmHr*, Hyg^R^ | This work |
| ppAE159-*sigF* | phAE159 containing ~900bp fragments up- and downstream of *sigF*, Hyg^R^ | (3) |
| pET*-msmHr* | pET23b(+) containing full length of *msmHr*, Amp^R^ | This work |

Amp^R^, Hyg^R^ and Km^R^ indicate resistance to ampicillin, hygromycin and kanamycin, respectively.

1. **Stover CK, de la Cruz VF, Fuerst TR, Burlein JE, Benson LA, Bennett LT, Bansal GP, Young JF, Lee MH, Hatfull GF, et al.** 1991. New use of BCG for recombinant vaccines. Nature **351:**456-460.

2. **Bardarov S, Bardarov Jr S, Jr., Pavelka Jr MS, Jr., Sambandamurthy V, Larsen M, Tufariello J, Chan J, Hatfull G, Jacobs Jr WR, Jr.** 2002. Specialized transduction: an efficient method for generating marked and unmarked targeted gene disruptions in Mycobacterium tuberculosis, M. bovis BCG and M. smegmatis. Microbiology **148:**3007-3017.

3. **Wu H, Hu X, Xiao J, Zhang J, Tao J, Huang H, Mi K.** 2012. [Sigma factor F regulates Mycobacterium smegmatis hydrogen peroxide resistance]. Wei sheng wu xue bao = Acta microbiologica Sinica **52:**1352-1359.

.
