# Supplementary material for: A bacterial hemerythrin-like protein MsmHr inhibits the SigF-dependent hydrogen peroxide response in mycobacteria
Source: Front Microbiol. 2015 Jan 15;5:800. doi: 10.3389/fmicb.2014.00800 (PMC4295536; doi:10.3389/fmicb.2014.00800)
Supplement: Supplementary file 2 [file Table2.DOC]

**Table S2. Oligonucleotide primers used in this study***

| Name | 5’-3’ | purpose |
| --- | --- | --- |
| Msm1804qF | CGGCTCAAGGAACTCCACTT | qRT-PCR |
| Msm1804qR | TCTTCGCGATCCATGTCCAG |
| MsmRpoDqF | GTGTGGGACGAGGAAGAGTC | qRT-PCR |
| MsmRpoDqR | ACCTCTTCTTCGGCGTTGAG |
| Msm1782qF | TGGCGGCACACATTCGCAGT | qRT-PCR |
| Msm1782qR | TTGCCGCGCAGTCCGTTGAT |
| Msm4753qF | CCGGGGCCGAAAGGTCATCG | qRT-PCR |
| Msm4753qR | CTCGGGCTTGTCGGGGGAGA |
| Msm2414qF | GTACTCGTTCGTGTCGTCGT | qRT-PCR |
| Msm2414qR | AACCGTTGAGCTTCTCCTGC |  |
| Msm2415qF | CAGACCGCTTTCGTCGAACT | qRT-PCR |
| Msm2415qR | GCTTGGCCTTGTGTTCCTCT |  |
| Msm2416qF | CTACGTCGACAGCAAACTGG | qRT-PCR |
| Msm2416qR | GGGTCACGTAGTCGTGTGTC |  |
| Msm2415pETF | ACGTGGATCCCCTCGGAGGATTCACCGTG | pET23b- *msmHr* |
| Msm2415pETR | GCGTAAGCTTCACGTCAGCGCTACTTCAG |
| Msm2415pMVF | AGCGGAATTCGTGGCCGATTCAAAGCCCA | pMV261- *msmHr* |
| Msm2415pMVR | ACGTGGATCCCACGTCAGCGCTACTTCAG |
| 2415H1MF | CAGCTGCTGGCGGTGCTCGAGACCGCCGCGGCGATGGTGGTGCACCCGAG | point mutation |
| 2415H1MR | CTCGGGTGCACCACCATCGCCGCGGCGGTCTCGAGCACCGCCAGCAGCTG |
| 2415H2MF | GCCTCGAAGAGGAACTCAAGGCCAAGGCGGCACTGTCCGAGCTCGAG | point mutation |
| 2415H2MR | CTCGAGCTCGGACAGTGCCGCCTTGGCCTTGAGTTCCTCTTCGAGGC |
| 2415H3MF | CTCGATCACGCCGCCCTCGAGGAGGCCGCGGCGTTCGTCAAGCTCTC | point mutation |
| 2415H3MR | GAGAGCTTGACGAACGCCGCGGCCTCCTCGAGGGCGGCGTGATCGAG |
| PsigFF | AAATTTGGATCCGGACAGCGGAATCGATACATGAAG | P*_rbsW_*-*lacZ* |
| PsigFR | AAATTTGGTACCCACCACCGCCAGGTTCTCCAAC |
| P1802F | AAATTTGGATCCGCTGCGGCGCCTTCTCATCTC | P*msmeg_1802*-*lacZ* |
| P1802R | AAATTTGGTACCGCGACGCTTCTTTGCCTCCCAGTG |
| P2415F | AAATTTGGATCCGTTCTGGTGAACCCCAACAAG | P*_Msmhr_*-*lacZ* |
| P2415R | AAATTTGGTACCCCGTGGACTACCCCCTACC |  |
| 2415LL | TTTTTTTTCCATAAATTGGTTCAACAGCGACCTCGTGAC | gene knockout |
| 2415LR | TTTTTTTTCCATTTCTTGGTGTGCTGATCGGTGAGAAAC |
| 2415RL | TTTTTTTTCCATAGATTGGATCGGCTCGGCCCTGAA | gene knockout |
| 2415RR | TTTTTTTTCCATCTTTTGGTTCGCATGCGTCGCCATA |
| 1804LL | TTTTTTTTCAGAAACTGAAGACGACCCGTGAGGGTA | gene knockout |
| 1804LR | TTTTTTTTCAGTTCCTGATGTCGAGAACGTCTGCGT |
| 1804RL | TTTTTTTTCAGAGACTGTGCTGGCCAAGTCGCT | gene knockout |
| 1804RR | TTTTTTTTCAGCTTCTGTGGGAACACGATGGAACCT |
| 2415LLL | TACGACGTCGACGCACGCAC | knockout identification |
| 2415RRR | CGATGAGATCCAAGCCGATTCGT |
| 2415InL | AACCTGATCAAGGACCTCTTCG | knockout identification |
| 2415InR | CGACGGCGAAGTTCAGCGA |
| 1804LLL | CGGACAGCGGAATCGATACATGAAGA | knockout identification |
| 1804RRR | ACCGTCATCGCCGAGATCAAGTA |
| 1804InL | GACAACCGAGGGTTCGCCGAGCT | knockout identification |
| 1804InR | GATTCGAAGAATCGCAGCACCAGCA |
| IL(R) | TCGACGACCCTAGAGTCC | knockout identification |
| IR(F) | GACACACCAACAGCATGGT |
| Msm6232qF | CGCAGGCAGAACCTCGGCAA | qRT-PCR |
| Msm6232qR | GGCCATCACCACGCGTTCGT |  |
| Msm6384qF | TGCCCGCGGAGTTCAAGCTG | qRT-PCR |
| Msm6384qR | GTTGACGTCGAGCACCCGCA |  |
| Msm6213qF | CCGCTTCCAGAACGTCGCGT | qRT-PCR |
| Msm6213qR | TGCGCTTCTCACCACCGAGC |  |
| Msm3101qF | CCGCTCGATGAGACCACGGC | qRT-PCR |
| Msm3101qR | AGGGTGATGGCCGCGTAGGT |  |
| Msm1808qF | GTCAGATCCCCAGGCCGTGC | qRT-PCR |
| Msm1808qR | GGAACCGCGAGGATGTCGGC |  |
| Msm3123qF | ACCACACTGCCGCTCACACG | qRT-PCR |
| Msm3123qR | TCGTAGGTGTCGGTGCCGGT |  |
| Msm2752qF | ATGCCACCACAAGCCGCGTT | qRT-PCR |
| Msm2752qR | CCTCGATGCGCTTGGCGAGT |  |
| Msm6199qF | GCATGACCGAACGCCAACGC | qRT-PCR |
| Msm6199qR | AGACTGCGCTGCGGTGCTTG |  |
| Msm3296qF | CGACTACCAACTCATGCCGT | qRT-PCR |
| Msm3296qR | ATCCGACATGTTCCGCATGG |  |
| Msm6467qF | GCGAGCGATGTGGCGGATCT | qRT-PCR |
| Msm6467qR | TGAAGTTCGGGCCCACGACG |  |
| Msm6896qF | GACGAGATCGGCCCGTCCCT | qRT-PCR |
| Msm6896qR | GCGCTACCCCACGGATCGTC |  |
| Msm5580qF | TCGATCCGCTGATGTACGAC | qRT-PCR |
| Msm5580qR | ATCATCATGACGTCGCGCTT |  |
| Msm2389qF | CACAAGGGCGATAGCGTGAC | qRT-PCR |
| Msm2389qR | CTGTGCGCCAGAGATAACCG |  |
| Msm0880qF | AAGCACCGCATCGAGGACGC | qRT-PCR |
| Msm0880qR | CACCGGTGAGCGACAGCTCC |  |
| Msm0711qF | CGAGGACGGTCAGCGCATCC | qRT-PCR |
| Msm0711qR | TCGCGCCCGAACACCTTGTC |  |
| Msm5696qF | CCTCAAGCTCATCGACCCG | qRT-PCR |
| Msm5696qR | GGACGACCTCCGAAACGC |  |

* Restriction enzyme sites and mutation sequences are underlined and double-underlined, respectively.
