# Supplementary material for: A bacterial hemerythrin-like protein MsmHr inhibits the SigF-dependent hydrogen peroxide response in mycobacteria
Source: Front Microbiol. 2015 Jan 15;5:800. doi: 10.3389/fmicb.2014.00800 (PMC4295536; doi:10.3389/fmicb.2014.00800)
Supplement: Supplementary file 3 [file Table3.DOCX]

Table S3 Quantitative assessment of the relatively mRNA level in wild-type and *msmHr* mutant following exposure to hydrogen peroxide

| Locus | Gene product | Relative fold expression after treatment H_2_O_2_    mc^2^155 Δ*msmHr* |
| --- | --- | --- |
| Redox related protein |  |  |
| MSMEG_6232 | Catalase KatA | 1.46±0.08 0.74±0.01 |
| MSMEG_6384 | Catalase-peroxidase KatG | 1.78±0.13 1.29±0.11 |
| MSMEG_1782 | Oxidoreductase, short chain dehydrogenase | 3.52±1.86 0.54±0.15 |
| MSMEG_4753 | Antioxidant, AhpC/TSA family protein | 5.63±0.91 2.82±0.65 |
| MSMEG_6213 | Mangnese containing catalase | 1.93±0.143 1.12±0.15 |
| MSMEG_3101 | Glucose-6-phosphate 1-dehydrogenase | 2.17±0.37 0.99±0.62 |
| MSMEG_1808 | Fe-S metabolism associated SufE | 1.51±0.06 0.82±0.49 |
| MSMEG_3123 | FeS assembly protein SufD | 2.11±0.11 1.12±0.56 |
| Regulator |  |  |
| MSMEG_2752 | RNA polymerase sigma factor SigB | 0.81±0.07 0.80±0.02 |
| MSMEG_6199 | Transcription factor WhiB | 2.03±0.28 1.18±0.11 |
| MSMEG_3296 | ECF-family protein sigma factor H | 2.90±0.53 1.10±0.75 |
| DNA binding protein |  |  |
| MSMEG_6467 | Starvation-induced DNA protecting protein | 1.14±0.15 0.94±0.09 |
| MSMEG_6896 | Single-stranded DNA-binding protein | 3.29±0.58 1.06±0.95 |
| MSMEG_5580 | DNA end-binding protein Ku | 1.34±0.43 0.92±0.14 |
| MSMEG_2389 | DNA-binding protein HU | 1.45±0.25 0.90±0.58 |
| Stress response protein |  |  |
| MSMEG_0880 | Molecular chaperone GroEL | 1.73±0.12 1.29±0.11 |
| MSMEG_0711 | Molecular chaperone DnaJ | 1.75±0.177 1.21±0.01 |
| MSMEG_5696 | Cold-shock DNA-binding domain-containing protein | 1.20±0.07 0.95±0.15 |
