# Supplementary material for: A bacterial hemerythrin-like protein MsmHr inhibits the SigF-dependent hydrogen peroxide response in mycobacteria
Source: Front Microbiol. 2015 Jan 15;5:800. doi: 10.3389/fmicb.2014.00800 (PMC4295536; doi:10.3389/fmicb.2014.00800)
Supplement: Supplementary file 4 [file Presentation1.PDF]

Figure S1. Generation of the Δ*sigF* *M. smegmatis* strain. A. The genomic organization of the *msmHr* gene locus. (Upper panel) Genes appeared as large arrows in their native orientation. Small arrows represented the forward and reverse primers used for PCR, and the sizes of the amplified products are indicated. The location and orientation of the hygromycin cassette were also indicated (Bottom panel). No PCR product using primers 1804InL and 1804InR to amplify the coding sequences of *sigF*, while the PCR products for upstream and downstream of *sigF* were amplified using the primer pairs 1804LLL/IL(R) and1804RRR/IR(F), respectively.
B. Effect of *msmHr* deletion on *sigF* expression. Quantitative real-time PCR (qRT-PCR) analysis of *sigF* transcription. The primer pairs 1804qF/1804qR were used for qRT-PCR. The results are shown as means ± standard deviations for three replicates.

Figure S2. Purification of MsmHr. M is protein marker. Lane 3 is the purification of MsmHr, which molecular size is about 24kD. Lane 1 and 2 is the whole lysate of pET23b-*msmHr* non-induced and induced by arabinose.

Figure S3. CD spectral analysis of Galectin. Blank control: 20 mM Tris-Cl buffer, pH 7.5 alone (clear circle); Galectin: 100 mg/L galectin in 20 mM Tris-Cl buffer, pH 7.5 (black circle). Measurements were obtained at room temperature. Images are representative of 3 independent experiments.
