# Supplementary figures and images for: A bacterial hemerythrin-like protein MsmHr inhibits the SigF-dependent hydrogen peroxide response in mycobacteria
Source: Front Microbiol. 2015 Jan 15;5:800. doi: 10.3389/fmicb.2014.00800 (PMC4295536; doi:10.3389/fmicb.2014.00800)

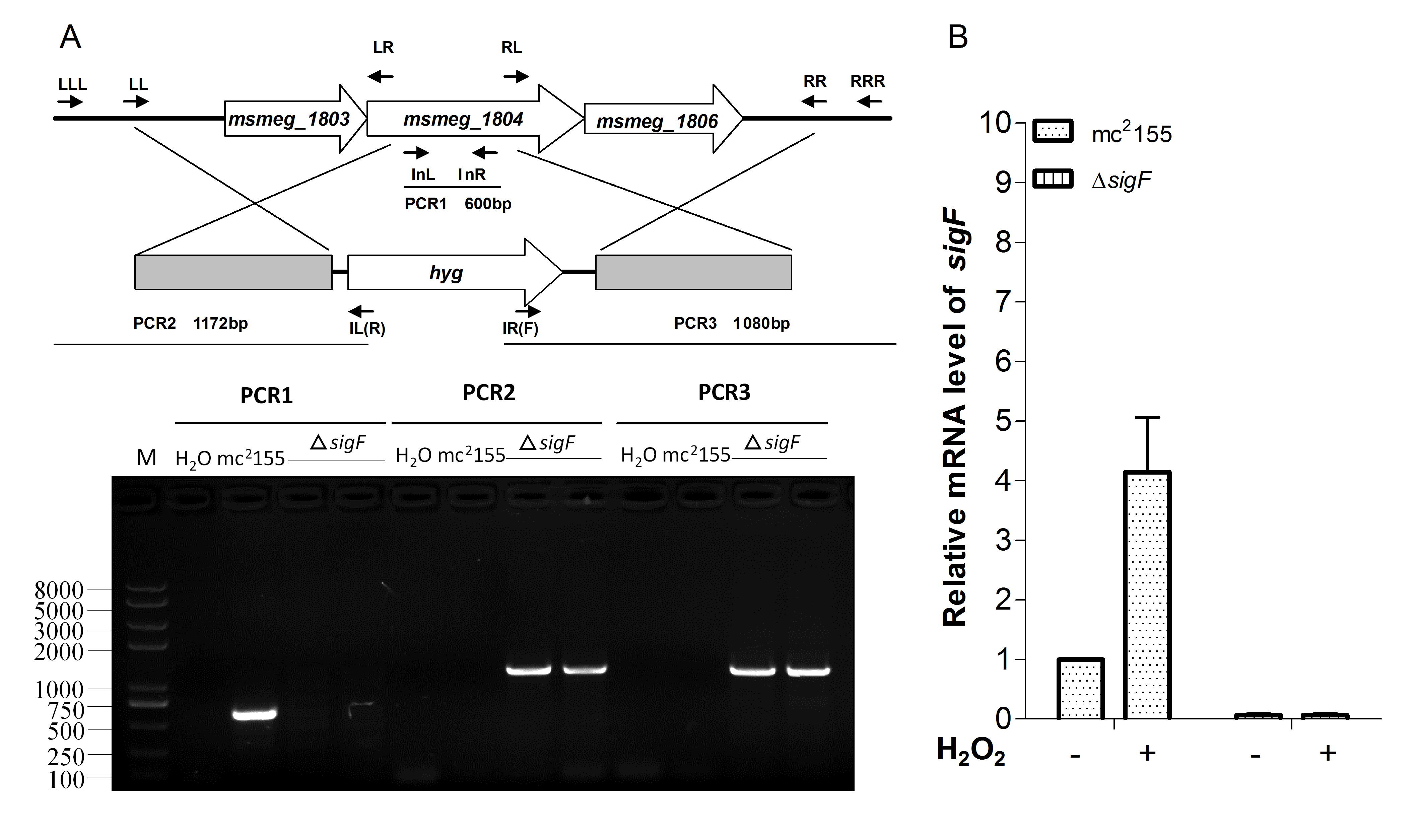

Supplement: Supplementary file 5 [file Image1.TIF]

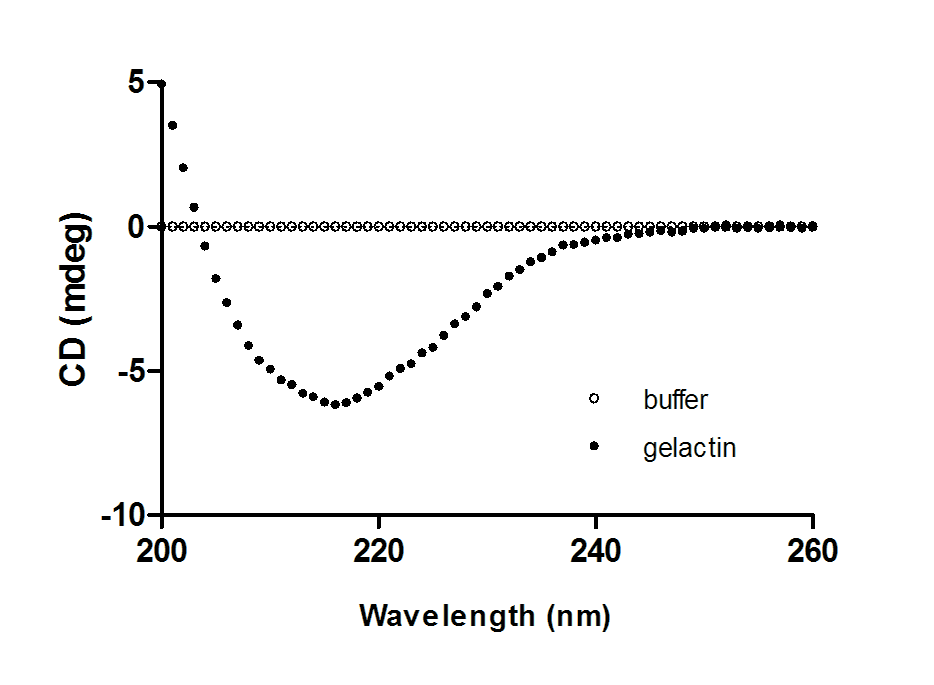

Supplement: Supplementary file 7 [file Image3.TIF]
